# Oncologic outcomes and prognostic factors of colloid carcinoma of the pancreas – a retrospective real-world data analysis from the German cancer registry group of the society of German tumor centers

**DOI:** 10.1007/s00423-025-03870-x

**Published:** 2025-09-23

**Authors:** Jannis Duhn, Lennart von Fritsch, Kim C. Honselmann, Louisa Bolm, Christoph Gerling, Kees Kleihues-van Tol, Maria Elena Lacruz, Constanze Schneider, Fabian Reinwald, Andrea Sackmann, Bianca Franke, Bernd Holleczek, Anna Krauß, Steffen Deichmann, Thaer S. A. Abdalla, Monika Klinkhammer-Schalke, Sylke Ruth Zeissig, Tobias Keck, Ulrich F. Wellner, Rüdiger Braun

**Affiliations:** 1 Department of Surgery, University Medical-Center Schleswig-Holstein, Campus Lübeck, Ratzeburger Allee 160, 23562 Lübeck, Germany; 2 Network for Care, Quality and Research in Oncology, German Cancer Registry Group of the Association of German Tumor Centers (ADT), Berlin, Germany; 3 Cancer Registry Saxony-Anhalt, Halle, Germany; 4 Clinical-epidemiological Cancer Registry Berlin-Brandenburg, Berlin, Germany; 5 Cancer Registry of Rhineland-Palatinate in the Institute for Digital Health Data, Mainz, Germany; 6 Hessian Cancer Registry, Frankfurt on the Main, Germany; 7 Cancer Registry Saarland, Saarbrücken, Germany; 8 Cancer Registry Mecklenburg-Western Pomerania, c/o Institute for Community Medicine, University Medicine Greifswald, Greifswald, Germany; 9 Institute of Clinical Epidemiology and Biometry (ICE-B), University of Würzburg, Würzburg, Germany; 10 Bavarian Cancer Registry, Bavarian Food and Health Safety Agency, Würzburg, Germany

**Keywords:** Pancreatic cancer, Pancreatic surgery, Colloid carcinoma, Cancer registry, Population based analysis, Real-world data

## Abstract

**Background:**

Colloid carcinoma (CC) is a rare subtype of pancreatic ductal adenocarcinoma (PDAC) characterized by mucin pools in over 80% of the tumor. This study compares the histopathology and outcomes of CC and not-otherwise specified PDAC (PDAC-NOS, referring to “classical” PDAC) using pooled data retrieved from regional cancer registries participating in the Clinical Cancer Registry Group of the Association of German Tumor Centers (GCRG/ADT).

**Materials and methods:**

Data from patients within the pooled dataset of the GCRG/ADT with pancreatic cancer (diagnosed between 2000 and 2023) were analyzed. Histological subtypes were identified by ICD-O3 histology code. Epidemiology, histopathology and survival rates were compared between surgically resected CC and PDAC-NOS. Prognostic impacts were assessed using uni- and multivariable regression analyses in R.

**Results:**

The study included 474 CC and 21,360 PDAC-NOS patients. CC patients presented more often without lymph node metastases (pN0: 44.0 vs. 31.5%, *p* < 0.001), lower grading and less lymphatic and blood vessel invasion (each *p* < 0.001). The R0 resection rate was similar in both groups. CC patients had superior OS compared to PDAC-NOS (median OS: 24.8 vs. 17.3 months, *p* < 0.001). Importantly, CC histology was an independent positive prognostic factor for OS. Grading, lymph- and blood-vessel invasion were independent prognostic factors for CC patients. Adjuvant therapy was associated with improved survival in UICC IIB CC patients.

**Conclusion:**

CC patients showed better oncological outcomes after surgical resection compared to PDAC-NOS. Thereby, CC-subtype is an independent positive prognostic factor for OS, associated with lower tumor stages, fewer lymph node metastases, and less vascular invasion.

**Supplementary Information:**

The online version contains supplementary material available at 10.1007/s00423-025-03870-x.

## Introduction

Pancreatic cancer is currently the fourth most common cause of cancer-related deaths and is predicted to become the second leading cause of cancer-related mortality by the year 2030 [1]. Pancreatic ductal adenocarcinoma (PDAC) is the most frequent histological subtype of malignancies of the exocrine pancreas [2]. Conventional PDAC refers to PDAC not otherwise specified (PDAC-NOS), i.e. not meeting criteria of a specific subtype defined by the current WHO definition.

Colloid carcinoma (CC) is a rare subtype of PDAC that accounts for only about 1% of all pancreatic tumors [3]. It is characterized by large pools of mucus that contain foci of malignant cells [4]. According to the WHO definition, CC is defined by mucin pools in > 80% of the tumor volume with scanty floating tumor cells [5, 6]. It has been postulated that the mucin functions as a physical barrier that inhibits spreading of the malignant cells and delays tumor progression [7, 8].

Although CC is defined by unique histopathological criteria as a subtype of PDAC, clinical symptoms are unspecific and comparable to those of conventional PDAC, i.e., abdominal or back pain, jaundice, and weight loss. There are no specific evidence-based treatment protocols for CC. Thus, CC treatment usually follows conventional PDAC treatment protocols.

Overall, the 5-year survival rate of PDAC patients remains as low as 12% ^9^. Most of the literature published on CC patients is limited to small retrospective case series. Khalil et al., however, recently reported a better clinical outcome of CC compared to PDAC-NOS based on a retrospective analysis of the National Cancer Database (NCDB) [10].

The aim of this study was to evaluate the pooled data regional cancer registries participating in the Cancer Registry Group of the Association of German Tumor Centers (GCRG/ADT). This registry database includes patients with pancreatic cancers treated at multiple regional certified German cancer centers between 2000 and 2023. Treatment regimens, clinical outcomes and prognostic factors for overall survival were analyzed for CC compared to PDAC-NOS patients.

## Materials and methods

### Study population

This retrospective study was approved by the ethics committee of the University of Luebeck, Germany (#2023 − 156) and performed in accordance with the data use regulations of the ADT. Population-based data retrieved from the nationwide Cancer Registry Group of the Association of German Tumor Centers (GCRG/ADT) was used for analysis. The GCRG/ADT regularly collects nationwide data from regional clinical cancer registries and establishes a pooled pseudonymized dataset. This dataset was queried for patients being diagnosed with pancreatic cancer (ICD-10: C25.0 – C25.9) between 2000 and 2023. Patients were further selected for histological subtype of CC (ICD-O-3 morphology code: 8480/3) and PDAC-NOS (ICD-O-3 morphology code: 8500/3 (invasive ductal carcinoma NOS) or 8140/3 (invasive ductal adenocarcinoma NOS), thereby representing classical PDACs excluding histologically otherwise defined subtypes).

### Study parameters

The following data were extracted for analysis: Age at diagnosis, sex, histopathological parameters.

(pT stage (pT0 – pT4), lymph node metastasis (pN0 – pN2), lymph- and blood vessel invasion (L/V0 – L/V1), grade of differentiation (G1 – G4) and resection margins (R0 – R2)), treatment regimens and chemotherapeutic agents used in adjuvant chemotherapy. Due to several changes in the TNM classification over the years (4th to 8th edition) [11] and pT3 were collectively categorized as pT2/3. For further analysis, several parameters were dichotomized as followed: pN0 versus pN+ (pN 1–2), R0 versus R+ (R 1–2) and G1/2 versus G3/4. Treatment regimens included: Surgical resection only, resection followed by adjuvant (radio-)chemotherapy, neoadjuvant (radio-)chemotherapy followed by resection, palliative (radio-)chemotherapy, neoadjuvant therapy without resection and palliative surgery. We further classified adjuvant chemotherapy regimens into Fluoropyrimidine-based (including FOLFIRINOX), Gemcitabine-based (including Gemcitabine + nab-Paclitaxel) and other regimens. The follow-up included time to follow-up (in months) from diagnosis and the status at last follow-up (alive/dead).

### Statistical analysis

Data processing and statistical analysis were performed using R (R Foundation for Statistical Computing, Vienna, Austria, version 4.4.2) with R Studio (Posit Software, PBC, Boston, USA). For descriptive statistics of continuous and categorical variables, median with interquartile range and absolute numbers with percentage of total were calculated, respectively. Differences between continuous variables were analyzed by two-sample Student’s t test. Dependencies of categorical variables were analyzed using Pearson’s Chi-squared test.

Two assess the overall survival, the Kaplan-Meier method and Log-rank test were used in the univariable analysis. A multivariable Cox proportional hazards model was fitted to assess the prognostic impact of independent variables. The significance level throughout this study was set to *p* < 0.05 (two-sided) and confidence intervals (CI) are reported as 95% CI.

## Results

### Patient cohort characteristics

Overall, 1700 patients with CC and 73,157 patients with PDAC-NOS diagnosed between 2000 and 2023 were identified and included (Suppl. Figure 1). Within the population of patients diagnosed with malignant pancreatic tumors (ICD10: C25.0 – C25.9), the prevalence of CC histology was 1.6%. In the study population consisting of CC and PDAC-NOS patients, the overall percentage of CC diagnosis was 2.3%. In both cohorts, CC was diagnosed more frequently before 2008, with frequencies remaining stable since (Suppl. Figure 2). The mean age (68.9 vs. 69.3 years) and sex (male: 54.5 vs. 53.4%) were comparable between CC and PDAC-NOS patients (Table 1a). CC patients showed significantly more often metastasis at diagnosis compared to PDAC-NOS patients (M1: 41.5 vs. 38.9%, *p* = 0.034). This results in tendency to fewer CC patients eligible for surgical resection (49.4 vs. 52.1%) compared to PDAC-NOS.

**Table 1:** A and b: demographics overall cohort and histopathology of the surgical cohort. The table displaying the demographical parameters of the overall cohort of patients with CC and PDAC-NOS tumor histology **(**Table 1a**)**, and demographical, histopathological and treatment courses in resected patients (Surgical cohort, Table 1b). Shown are total numbers with percentages in brackets. Categorical variables were compared by Chi-squared test and continuous variables by unpaired T test. Missing values were not included in the statistical analysis

Parameter	*N* (% of total)/mean (SD)		*p*- value
	CC	PDAC-NOS	
a. All patients			
Total number	1700	73,157	
Sex			0.356
male	927 (54.5)	39,043 (53.4)	
female	773 (45.5)	34,112 (46.6)	
*Missing data*	*0 (0.0)*	*2 (< 0.1)*	
Mean age (years)	68.9 (10.1)	69.3 (10.3)	0.071
Distant metastases			0.034
M0	995 (58.5)	44,692 (61.1)	
M1	705 (41.5)	28,465 (38.9)	
*Missing data*	*0 (0)*	*0 (0.0)*	
Treatment			0.066
(Radio-)chemotherapy	471 (49.1)	19,289 (45.1)	
Resection only	233 (24.3)	10,801 (25.2)	
Resection + adjuvant Therapy	222 (23.1)	10,491 (24.5)	
Neoadjuvant treatment + resection	19 (2.0)	1017 (2.4)	
Neoadjuvant treatment without resection	15 (1.6)	1143 (2.7)	
Palliative surgery + (radio-)chemotherapy	0 (0.0)	43 (0.1)	
*Missing data*	*740 (43.5)*	*30,373 (41.5)*	
b. Surgically resected patients			
Total number	474	21,360	
Sex			0.005
male	271 (59.0)	11,166 (52.3)	
female	188 (41.0)	10,193 (47.7)	
*Missing data*	*15 (3.2)*	*1 (< 0.1)*	
Mean age (years)	68.7 (9.3)	68.6 (9.8)	0.804
Tumor Localization			0.001
Pancreatic head	321 (69.9)	16,317 (76.4)	
Pancreatic body	31 (6.8)	1632 (7.6)	
Pancreatic tail	47 (10.2)	1668 (7.8)	
Pancreas multiple locations	27 (5.9)	839 (3.9)	
Pancreas NOS	33 (7.2)	904 (4.2)	
*Missing data*	*15 (3.2)*	*0 (0.0)*	
Surgical procedure			0.026
Partial pancreatoduodenectomy	298 (67.9)	14,864 (73.1)	
Distal pancreatectomy	73 (16.6)	3099 (15.2)	
Total pancreatectomy	68 (15.5)	2383 (11.7)	
*Missing data*	*35 (7.4)*	*1014 (4.7)*	
Pathological classification of resected tumor			
Tumor stage			0.151
pT0	1 (0.2)	45 (0.2)	
pT1	38 (8.5)	1428 (6.8)	
pT2/T3	391 (87.7)	18,848 (90.0)	
pT4	16 (3.6)	626 (3.0)	
*Missing data*	*28 (5.9)*	*413 (1.9)*	
Lymph node status			< 0.001
pN0	196 (44.0)	6578 (31.5)	
pN+	249 (56.0)	14,293 (68.5)	
*Missing data*	*29 (6.1)*	*519 (2.4)*	
Grading			< 0.001
G1	49 (12.1)	906 (4.6)	
G2	226 (55.9)	10,188 (52.1)	
G3	129 (31.9)	8397 (42.9)	
G4	0 (0.0)	66 (0.3)	
*Missing data*	*70 (14.8)*	*1803 (8.4)*	
Lymphatic vessel invasion			0.001
L0	179 (54.1)	7462 (44.4)	
L1	152 (45.9)	9333 (55.6)	
*Missing data*	*143 (30.2)*	*4565 (21.4)*	
Blood vessel invasion			< 0.001
V0	272 (85.8)	12,510 (76.0)	
V1	45 (14.2)	3958 (24.0)	
*Missing data*	*157 (33.1)*	*4892 (22.9)*	
Resection status			0.647
R0	229 (72.9)	11,148 (71.6)	
R+	85 (27.1)	4424 (28.4)	
*Missing data*	*160 (33.8)*	*5788 (27.1)*	
Patients treated with adjuvant chemotherapy			0.686
Resection + adjuvant therapy	214 (48.0)	10,061 (49.1)	
Resection only	232 (52.0)	10,445 (50.9)	
*Missing data*	*28 (5.9)*	*854 (4.0)*	
Adjuvant Chemotherapy Regimen			0.858
Gemcitabine-based	95 (79.8)	4641 (77.9)	
FOLFIRINOX	14 (11.8)	844 (14.2)	
FOLFOX	1 (0.8)	31 (0.5)	
Capecitbine-based	9 (7.6)	441 (7.4)	
*Missing data*	*95 (44.4)*	*4501 (44.7)*	

For further analysis, all patients that received surgical resection in the absence of distant metastasis (cM0 status), with or without (neo-)adjuvant treatment, were selected. Thus, 459 resected CC and 21,360 resected PDAC-NOS patients were included. CC patients were more frequently treated by total pancreatectomy (15.5 vs. 11.7%, *p* = 0.026) (Table 1b) and less often by pancreatoduodenectomy (67.9 vs. 73.1%) than PDAC-NOS patients. This is likely explained by more frequent multifocal location of CC compared to PDAC-NOS (5.9 vs. 3.9%, *p* = 0.001).

After surgical resection, 48.8 and 49.1% of patients with CC or PDAC-NOS were treated with adjuvant therapy (*p* = 0.686), respectively. The chemotherapy regimens used were comparable between both groups, with most patients treated by Gemcitabine-based chemotherapy (79.8 vs. 77.9%, *p* = 0.858), followed by FOLFIRINOX regimens (11.8 vs. 14.2%), although high-rates of missing data may affect the evaluation.

### Histopathological analysis of resected tumors

Resected tumors with CC compared to PDAC-NOS histology tended to be smaller in tumor size (pT1: 8.5 vs. 6.8%, *p* = 0.151) and showed less lymph node metastasis (pN0: 44.0 vs. 31.5%, *p* < 0.001). In addition, lymphatic (L0: 54.1 vs. 44.4%, *p* = 0.001) and blood vessel invasion (V0: 85.8 vs. 76.0%, *p* < 0.001) was less frequent in CC tumors compared to PDAC-NOS (Table 1b). In addition, CC tumors showed a higher grade of differentiation (G1/2: 68.0 vs. 56.7%, *p* < 0.001). However, besides presenting at less advanced tumor stages, the R0 resection rate was comparable between both groups (R0: 72.9 vs. 71.6%, *p* = 0.647).

### Survival of resected CC patients compared to PDAC-NOS

**Fig. 1:**
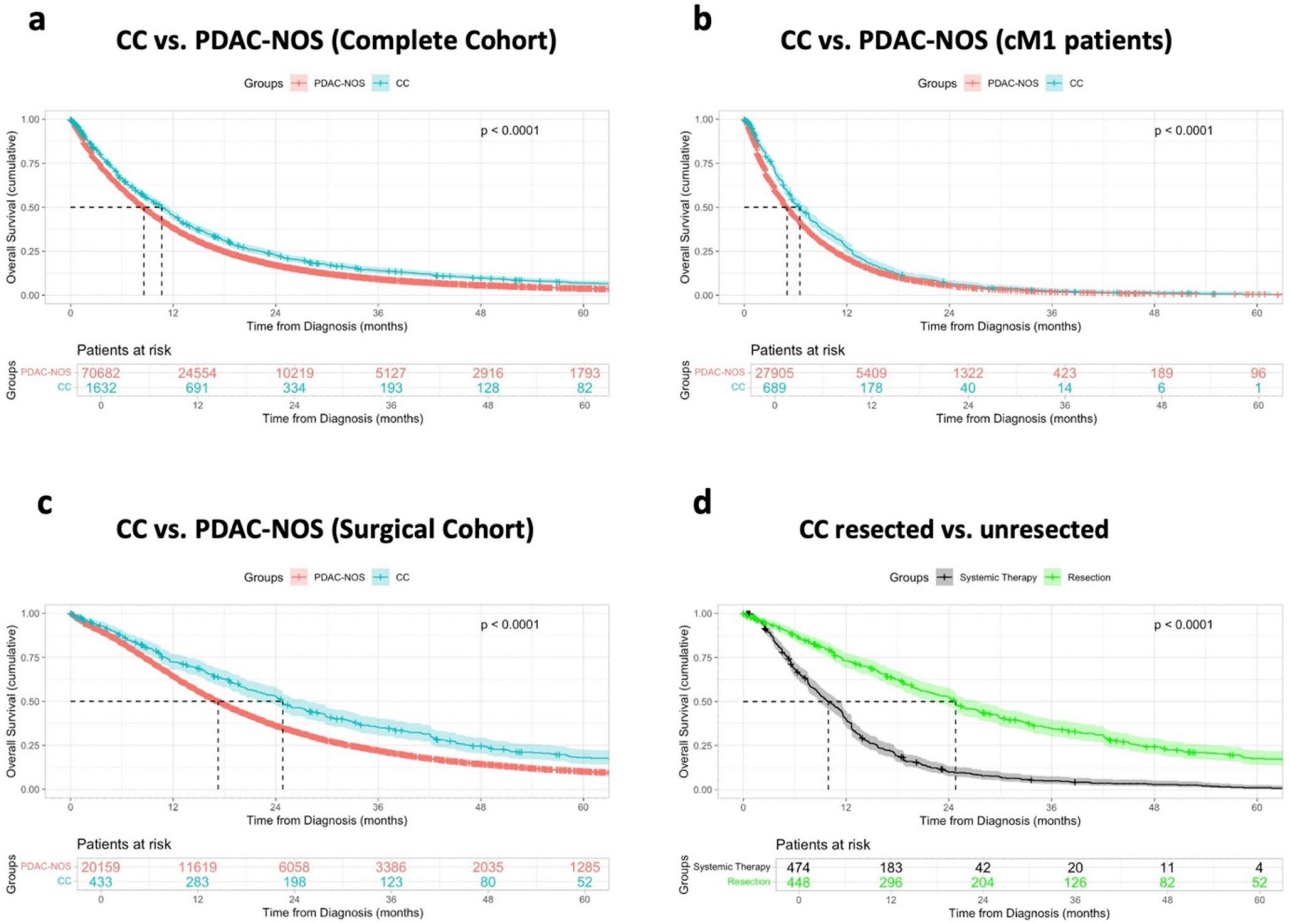
Survival Analysis. Kaplan-Meier curves showing the overall survival from diagnosis in months with 95% Confidence interval of all patients diagnosed with CC and PDAC-NOS **a**, and CC and PDAC-NOS patients with cM1 status at diagnosis independent of the treatment regimen **b**. Comparison of survival in resected CC and PDAC-NOS patients **c** and resected vs. unresected CC patients **d**. The displayed p-value values calculated using the Log-rank test

Overall, CC patients had superior overall survival (OS) from time of diagnosis compared to PDAC-NOS patients (median OS: 10.7 vs. 8.6 months, *p* < 0.001, Fig. 1a), despite presenting with more frequent M1 status at diagnosis. In the M1 group, CC patients also showed improved OS over PDAC-NOS patients (median OS: 6.5 vs. 5.0 months, *p* < 0.001, Fig. 1b). In the surgically resected cohort, CC patients showed a significantly better OS compared to PDAC-NOS patients (median OS: 24.8 vs. 17.3 months, *p* < 0.001, Fig. 1c). Patients who underwent surgical resection showed significantly improved OS compared to (radio-)chemotherapy alone, independent of the histology (Fig. 1d).

**Fig. 2:**
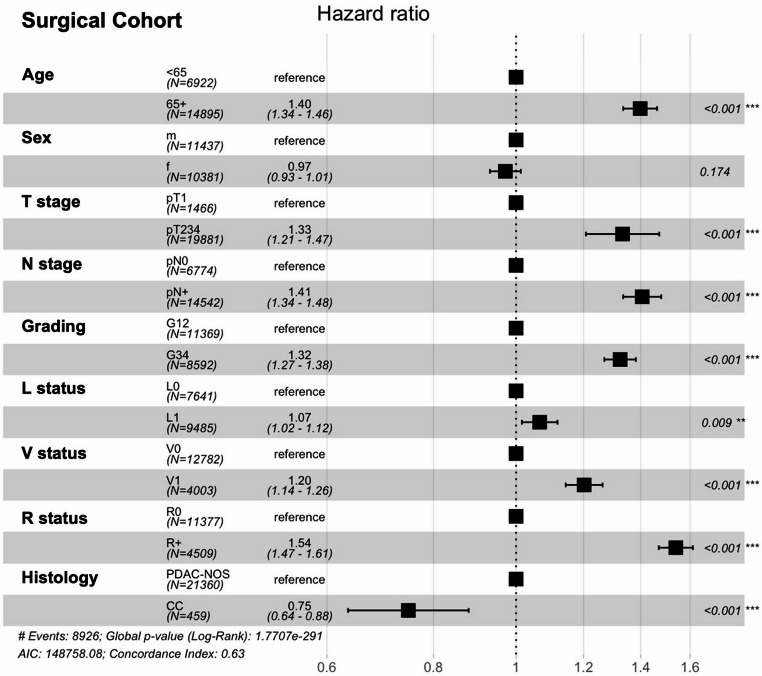
Multivariable Analysis Surgical Cohort. Multivariable cox-regression analysis of the overall survival from time of diagnosis of the complete Surgical cohort (CC + PDAC-NOS). Shown are the hazards ratios with the 95% Confidence Interval, as well as the p-values

To further evaluate prognostic factors after surgical resection, a multivariable Cox proportional hazards model was fitted in the combined cohort of resected CC and PDAC-NOS patients (Fig. 2). Age ≥ 65 (HR 1.37, *p* < 0.001) was associated with worse OS. Regarding histopathological parameters, larger tumor size (pT2–4 vs. pT1), presence of lymph node metastasis (pN + vs. pN0), higher grading (G3–4 vs. G1–2), lymph and blood vessel invasion, and positive resection margins (R + vs. R0) were negative prognostic factors (each *p* < 0.05). Importantly, CC histology was an independent positive prognostic factor for OS (HR 0.75, *p* < 0.001).

In addition, patients with 8th edition AJCC staging were identified and stratified according to UICC I, II and III stage and compared regarding the OS. Thereby, CC patients showed improved OS in UICC stage I patients over PDAC-NOS, whereas no significant differences were identified in UICC stage II and III (Suppl. Figure 3). However, samples sizes were small in these analyses.

### Prognostic factors in resected CC patients

**Fig. 3:**
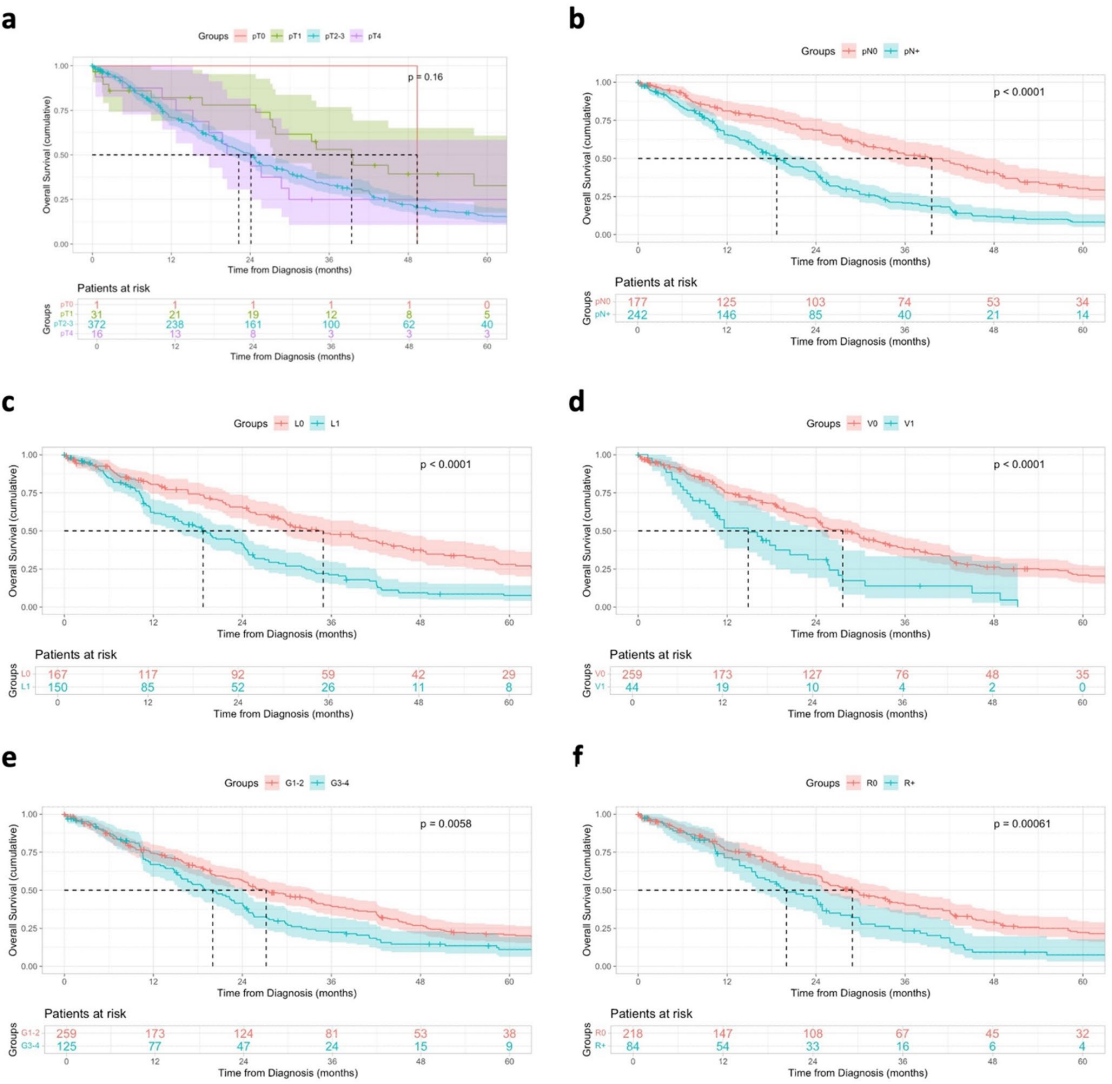
Survival Analyses of Prognostic Features in CC. Kaplan-Meier curves showing the overall survival from diagnosis in months with 95% Confidence interval of resected CC patients. The displayed p-values were calculated using the Log-rank test

**Fig. 4:**
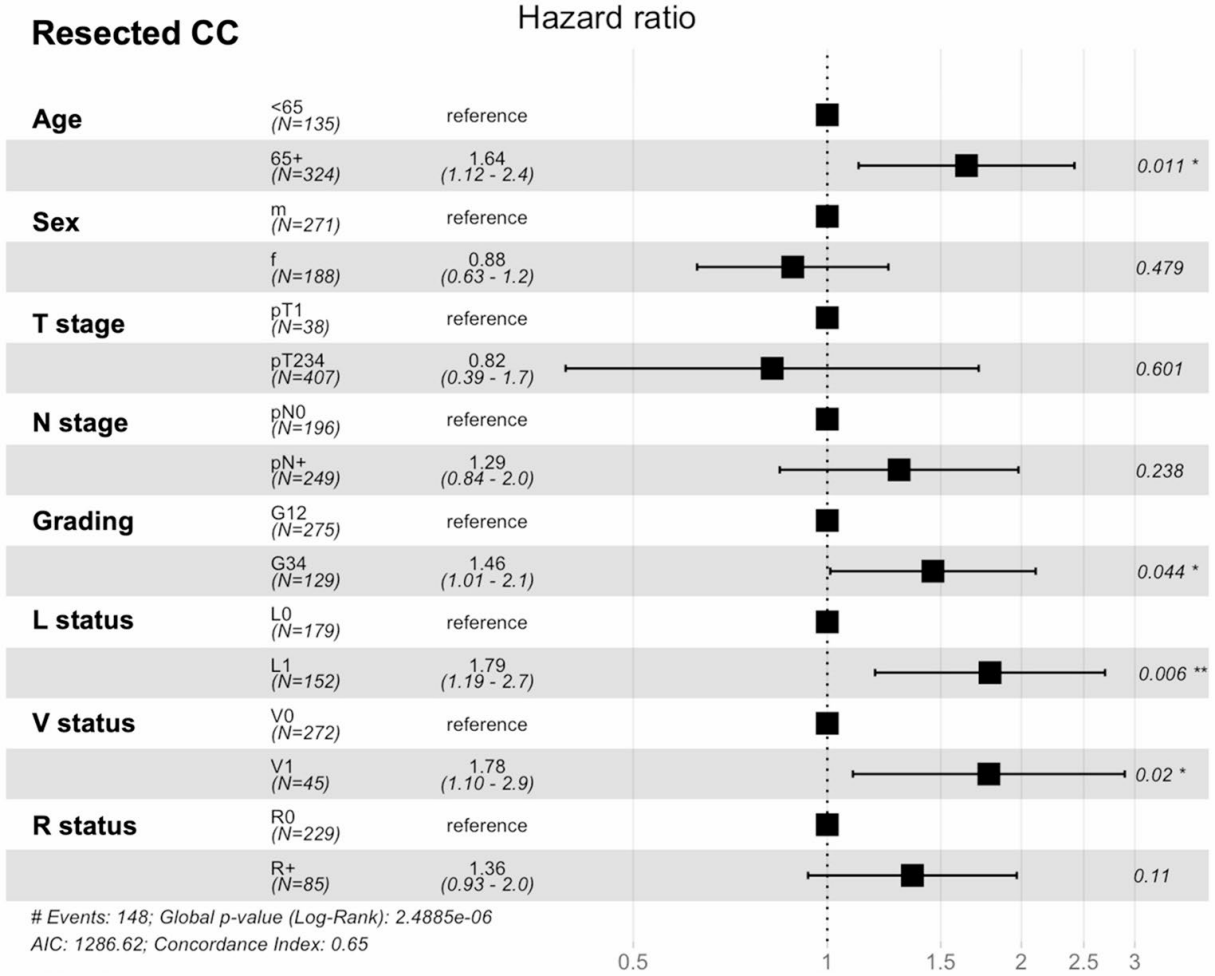
Multivariable analysis prognostic factors in resected CC patients. Multivariable cox-regression analysis of the overall survival of resected CC patients. Shown are the hazards ratios with the 95% Confidence Interval, as well as the p-values

Univariable analysis (Kaplan-Meier plot and Log-rank test) were performed to confirm prognostic factors in resected patients with CC. Age ≥ 65 was associated with worse OS (*p* = 0.014), in contrast sex did not affect OS (*p* = 0.73) (Suppl. Table 1a). Higher T stage was not significantly associated with OS (*p* = 0.16), lymph node metastasis (*p* < 0.001), lymphangioinvasion (*p* < 0.001) and hemangioinvasion (*p* < 0.001) were identified as negative prognostic factors (Fig. 3, Suppl. Table 1b). In addition, OS was significantly worse in patients with tumor positive resection margins (*p* < 0.001) and undifferentiated tumors (*p* = 0.005). In multivariable analysis, age ≥ 65 years (HR 1.64, *p* = 0.011), tumor Grading 3/4 (HR 1.46, *p* = 0.044), lymphangioinvasion (HR 1.79, *p* = 0.006) and hemangioinvasion (HR 1.78, *p* = 0.02) were identified as independent negative prognostic factors (Fig. 4). The identified prognostic factors were comparable for surgically resected PDAC-NOS patients (Suppl. Table 1a-b, Suppl. Figure 4).

**Fig. 5:**
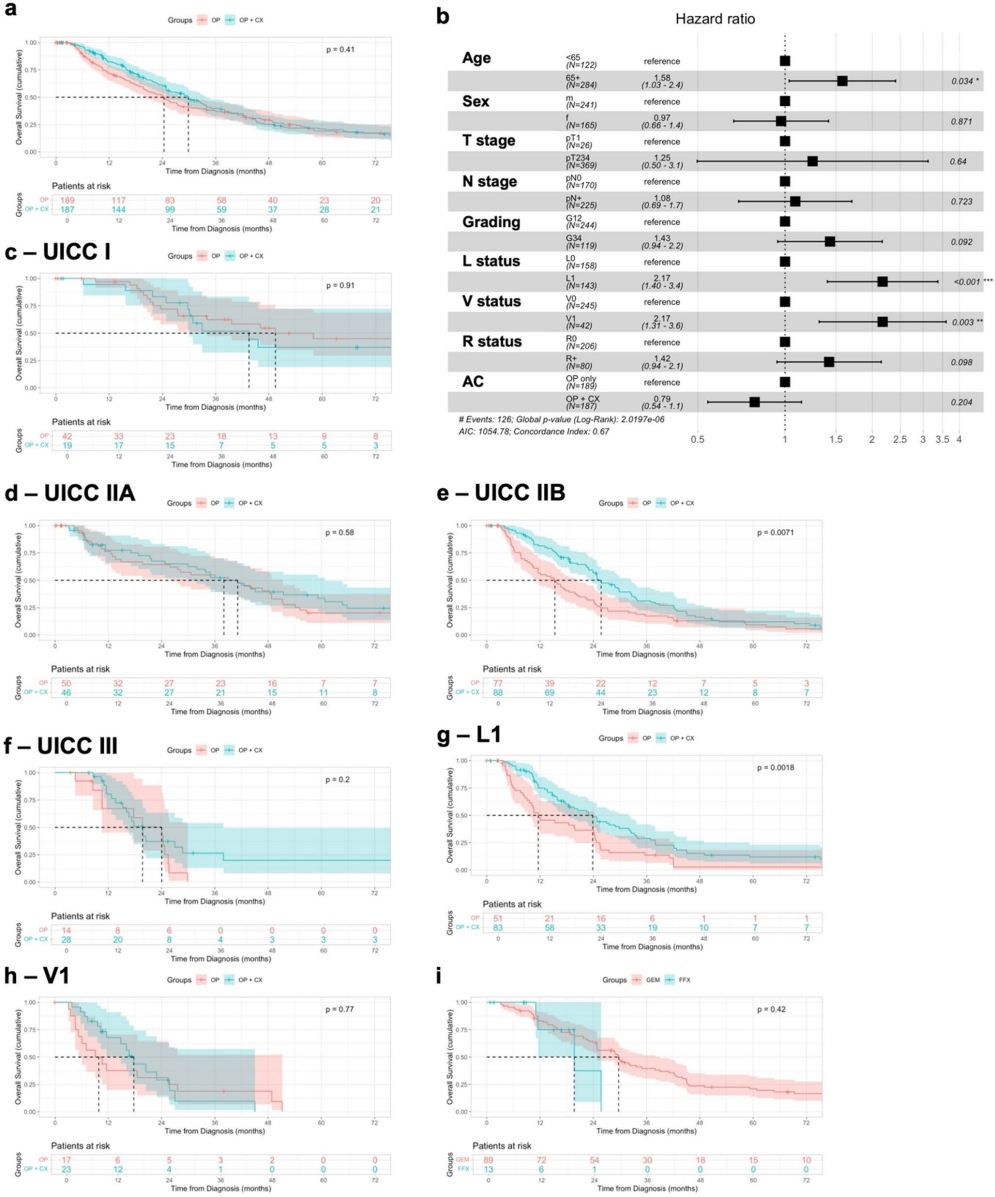
Analysis of adjuvant chemotherapy in CC patients. Association of adjuvant chemotherapy in CC patients after exclusion of patients with 90-day postoperative mortality (**a**,** b**), and subgroup analysis of patients stratified according to pathological UICC stages and negative prognostic factors (**c-h**). Comparison of Gemcitabine (GEM)- and FOLFIRINOX (FFX)-based adjuvant chemotherapy (**i**). Kaplan-Meier curves showing the overall survival from diagnosis in months with 95% Confidence interval, the displayed p-values were calculated using the Log-rank test. Forest plots showing the hazards ratios with the 95% Confidence Interval, as well as the p-values following a multivariable Cox-regression

The effect of adjuvant therapy was evaluated after exclusion of patients with 90-day postoperative mortality, to prevent a retrospective selection bias. Thereby, adjuvant therapy did not improve OS in univariable analysis (median OS: 29.8 vs. 24.2 months, *p* = 0.35, Fig. 5a, Suppl. Table 2) and after adjustment for confounding variables in a multivariable regression analysis (HR 0.79, *p* = 0.204, Fig. 5b). Interestingly, we were observed that adjuvant therapy was associated with improved survival in CC patients with nodal positive disease (UICC stage IIB) or lymphangioinvasion, but not in other subgroups (Fig. 5c-h). In a small subgroup analysis of patients with documented chemotherapy regimen, there was no difference in OS depending on treatment with FOLFIRINOX (FFX) or Gemcitabine-based therapies (GEM) (Fig. 5i).

## Discussion

Pancreatic cancer still has a limited prognosis despite recent improvements in surgical and radiation techniques, as well as in systemic treatments [9]. Despite this dismal prognosis, some rare histopathological subtypes of pancreatic cancer have a superior prognosis and survival but are not well characterized in the literature. Colloid Carcinoma (CC) has been described as one of the rare histopathological subtypes of PDAC and is defined by mucin pools in > 80% of the tumor volume with scanty floating tumor cells [5]. Intraductal papillary mucinous neoplasms (IPMN), which are defined as a mucin-producing cystic mass originating from the pancreatic ducts, can progress to two forms of invasive carcinoma, i.e. PDAC-NOS or CC [12]. The latter commonly arise from intestinal type IPMN and can be differentiated from PDAC-NOS by muconodular invasion type [13]with scant tumor cell clusters, and by their expression of the intestinal epithelial marker MUC2 and intestinal differentiation marker CDX2 [12, 14]. Based on these criteria, PDAC-NOS and CC were given different ICD-O3-morphology classification codes (PDAC-NOS: 8500/3 or 8140/3, CC: 8480/3) [15]. CC are typically diagnosed on histopathological work-up after surgical resection rather than on the preoperative diagnostic work-up due to their rare occurrence. In addition, the clinical presentation of CC and PDAC is very similar. Yet, there are no specific clinical consensus guidelines for treatment of CC in distinction to PDAC-NOS treatment.

Clinical data on CC is sparse and based on case reports or small case series [16]. Recently, Khalil et al. reported an analysis of the National Cancer Database (NCDB) that specifically analyzed characteristics and clinical outcomes of CC in comparison to PDAC-NOS [10]. The study cohort included 2430 CC patients and 54,416 PDAC patients between 2004 and 2016. There was no significant difference in sex and age distribution between CC and PDAC patients. Patients with CC compared to PDAC presented significantly more often with pathological stage I disease with 16.7% and 5.9%, respectively. In turn, stage IV disease was diagnosed less often (42.1% vs. 52.2%). Stage I disease CC patients received neoadjuvant and adjuvant chemotherapy significantly less frequently compared to PDAC patients. Strikingly, Khalil et al. found a significant improved overall survival of CC compared to PDAC patients in stages I, II and IV.

These data are in line with our analysis of nation-wide pooled clinical cancer registry data from Germany. The incidence of CC among all pancreatic cancer patients was 1.6%, which is in good agreement with previously reported incidence of 1–3% ^7^. CC was diagnosed more frequently before 2008, potentially suggesting more differentiated diagnostic procedures afterwards, leading to distinguishment of different subtypes. A similar trend was also observed by Khalil et al. in the NCDB database [10]. Therefore, an overestimation of CC diagnosis before 2008 cannot be fully excluded. In addition, instutional differences in diagnostic procedures may have played a role, which cannot be traced due to the nationwide structure of the cancer registry. Interestingly, stage IV disease was more frequent in CC than in PDAC-NOS patients, which is contrary to the findings of Khalil et al. However, subtype diagnosis based only on biopsies rather than resection specimen must be interpreted with great care, especially regarding the diagnostic criterium of mucin pools in > 80% of the tumor volume [5]. In addition, no information on the method of diagnosis (biopsy, type of biopsy vs. diagnosis based on imaging and type of imaging) is included in the registry dataset. Therefore, misdiagnosis of CC in non-resected patients cannot be ruled out, leading to potential overestimation in non-resected patients. To prevent this bias, we concentrated on resected patients in which diagnosis was based on histology of the resected specimen.

In line with the findings of Khalil et al., resected CC tended to be more often pT1 stage disease (8.5 vs. 6.5%, *p* = 0.614) and showed significantly less lymph node metastases (pN0: 44.0 vs. 31.5%, *p* < 0.001) compared to PDAC-NOS. In addition, CC had lower grading and less lymphatic as well as blood vessel invasion (*p* < 0.001). The R0 resection rate was comparable between CC and PDAC-NOS patients. In agreement with the data reported by Khalil et al., CC was associated with better OS compared to PDAC-NOS (median OS: 24.8 vs. 17.3 months, *p* < 0.001). Importantly, CC histology remained an independent positive prognostic factor (HR 0.75, *p* < 0.001) after adjustment for potentially confounding covariables in a multivariable regression. In resected CC patients, grading, lymph- and blood vessel invasion were proven to be independent prognostic factors in multivariable analysis.

CC patients tended to more frequently require a total pancreatectomy, as compared to PDAC-NOS, which might indicate a more multifocal distribution of these tumors in the pancreas. This might be explained to their development from intestinal type IPMNs [12].

It has been suggested that the favorable prognosis of patients with CC compared to PDAC-NOS may be due to altered cell biology. CC cells seem to have an inverse polarization in which the basal part of the cells secrets mucin towards the interface between the cells itself and the stroma, which separates the cell from the stroma [13, 16]. Potentially, mucin that surrounds the cancer cells prevents their spreading [13]. In the overall cohort, despite significantly higher incidence of M1 status resulting in palliative systemic therapy in CC compared to PDAC, the overall survival is superior in CC patients, further strengthening the idea of protective biological properties of CC tumors. Also, UICC IV patients with CC histology showed improved OS over PDAC-NOS. Therefore, a higher incidence of cM1 status at diagnosis in CC patients seems counter-intuitive, hence these results must be interpreted with care, especially regarding potential diagnostic issues.

As specific recommendations on treatment of patients with CC are lacking, most patients are treated in analogy to PDAC-NOS. Thereby, both German and American guidelines recommend adjuvant chemotherapy irrespective of the tumor stage in patients undergoing curative intent resection [17, 18]. However, our data show that only about 50% of all patients receive adjuvant chemotherapy, with rates being comparable in PDAC-NOS and CC patients. This is in line with findings from a cross-validation study using German (ADT) and American Cancer Registry (NCDB) based data, showing achievement of composite perioperative textbook outcome (R0 resection, ≥ 12 harvested lymph nodes, perioperative therapy) in only 50–54% of all patients with resected PDAC [19]. Interestingly, we observe a higher rate of administration of adjuvant chemotherapy in CC patients compared to results from the NCDB [10].

The positive effect of adjuvant chemotherapy in PDAC has been well described [20, 21]. However, the few existent studies reporting outcomes after adjuvant chemotherapy in CC yield controversial results. Picado et al. retrospectively evaluated the effect of perioperative chemoradiation in CC patients using the NCDB database, showing improvement of OS in patients with lymph-node positive disease (Stage IIB), but not in earlier stages [22]. Likewise, we were not able to observe an improved survival in patients with CC receiving adjuvant therapy over resection only, neither in a univariable analysis, nor after adjustment for confounding factors in a multivariable regression. We also conducted subgroup analyses in patients stratified according to pathological UICC stages. Like Picado et al. [22]we observed that adjuvant therapy is associated with improved survival in UICC IIB patients, but not other UICC stages. In addition, we observed improved survival following adjuvant therapy in patients with lymphangioinvasion. In contrast, reception of Gemcitabine or FOLFIRINOX-based chemotherapy was not significantly associated with survival. However, potential therapy protocols, dosages, and amounts of cycles received might have been highly heterogeneous, limiting the validity of this analysis. This discrepancy in outcomes following adjuvant chemotherapy has also been observed in other mucin-containing pancreatic tumors, like invasive IPMN, showing no survival benefit of adjuvant chemotherapy in early tumor stages [23–26]. These results emphasize the relevance of accurate histological tumor classification for further treatment stratification of patients following resection.

There is consensus that clinical cancer registry data gives important insights to the real-world situation of epidemiology, treatment, and survival of specific pancreatic cancer subtypes such as CC. Nonetheless, one must acknowledge that registry-based studies are limited by their retrospective design and missing data without possibility of source-validation. In addition, histological diagnosis based on biopsy in patients with systemic disease not undergoing surgical resection is less reliable, which might limit the validity of the overall cohort.

## Conclusions

This study is, to our knowledge, the first population-based analysis of cancer registry data outside the United States reporting treatment, histopathological data, and clinical patient outcomes of CC patients in comparison to PDAC-NOS, validating the results from the NCDB database recently published by Khalil et al. [10]. Our data shows less advanced tumors by means of less lymph node metastases, lower grading and less lymphatic and blood vessel invasion in CC tumors compared to PDAC-NOS. CC was proven as independent positive prognostic factor. Overall survival of resected CC patients was significantly improved in comparison to resected PDAC-NOS patients, highlighting the relevance of rare histological subtypes in PDAC. Adjuvant chemotherapy was associated with improved survival in patients in UICC IIB stage and with presence of lymphangioinvasion only.

## Supplementary Information

Below is the link to the electronic supplementary material.

ESM 1 (DOCX 20.6 KB)

ESM 2 (DOCX 744 KB)

## Data Availability

No datasets were generated or analysed during the current study.

## References

[1] Rahib L, Smith BD, Aizenberg R, Rosenzweig AB, Fleshman JM, Matrisian LM (2014) Projecting cancer incidence and deaths to 2030: the unexpected burden of thyroid, liver, and pancreas cancers in the United States. Cancer Res 74(11):2913–2921. 10.1158/0008-5472.CAN-14-0155 24840647 10.1158/0008-5472.CAN-14-0155

[2] Kleeff J, Korc M, Apte M et al (2016) Pancreatic cancer. Nat Rev Dis Primers 2:16022. 10.1038/nrdp.2016.22 27158978 10.1038/nrdp.2016.22

[3] Gao Y, Zhu YY, Yuan Z (2015) Colloid (mucinous non-cystic) carcinoma of the pancreas: a case report. Oncol Lett 10(5):3195–3198. 10.3892/ol.2015.3733 26722311 10.3892/ol.2015.3733 PMC4665270

[4] Whang EE, Danial T, Dunn JC et al (2000) The spectrum of mucin-producing adenocarcinoma of the pancreas. Pancreas 21(2):147–151. 10.1097/00006676-200008000-00007 10975708 10.1097/00006676-200008000-00007

[5] Bosman FT, Carneiro F, Hruban RH, Theise ND (2010) *WHO classification of tumours of the digestive system*. (F. T. Bosman FC RH Hruban, Theiseeditors ND (ed)). World Health Organization

[6] Nagtegaal ID, Odze RD, Klimstra D et al (2020) The 2019 WHO classification of tumours of the digestive system. Histopathology 76(2):182–188. 10.1111/his.13975 31433515 10.1111/his.13975 PMC7003895

[7] Liszka L, Zielinska-Pajak E, Pajak J, Gołka D (2008) Colloid carcinoma of the pancreas: review of selected pathological and clinical aspects. Pathol (Phila) 40(7):655–663. 10.1080/00313020802436444 10.1080/00313020802436444 18985519

[8] Bausch D, Mino-Kenudson M, Fernández-Del Castillo C, Warshaw AL, Kelly KA, Thayer SP (2009) Plectin-1 is a biomarker of malignant pancreatic intraductal papillary mucinous neoplasms. J Gastrointest Surg Off J Soc Surg Aliment Tract 13(11):1948–1954 discussion 1954. 10.1007/s11605-009-1001-9 10.1007/s11605-009-1001-9 PMC3806105 19760374

[9] Siegel RL, Miller KD, Wagle NS, Jemal A (2023) Cancer statistics, 2023. CA Cancer J Clin 73(1):17–48. 10.3322/caac.21763 36633525 10.3322/caac.21763

[10] Khalil L, Huang Z, Zakka K et al (2023) Survival and prognostic factors in patients with pancreatic colloid carcinoma compared with pancreatic ductal adenocarcinoma. Pancreas 52(1):e75–e84. 10.1097/MPA.0000000000002227 37378903 10.1097/MPA.0000000000002227 PMC10310320

[11] Chun YS, Pawlik TM, Vauthey JN (2018) 8th edition of the AJCC cancer staging manual: pancreas and hepatobiliary cancers. Ann Surg Oncol 25(4):845–847. 10.1245/s10434-017-6025-x 28752469 10.1245/s10434-017-6025-x

[12] Adsay V, Mino-Kenudson M, Furukawa T et al (2016) Pathologic evaluation and reporting of intraductal papillary mucinous neoplasms of the pancreas and other tumoral intraepithelial neoplasms of pancreatobiliary tract: recommendations of Verona consensus meeting. Ann Surg 263(1):162–177. 10.1097/SLA.0000000000001173 25775066 10.1097/SLA.0000000000001173 PMC4568174

[13] Adsay NV, Merati K, Nassar H et al (2003) Pathogenesis of colloid (pure mucinous) carcinoma of exocrine organs: coupling of gel-forming mucin (MUC2) production with altered cell Polarity and abnormal cell-stroma interaction May be the key factor in the morphogenesis and indolent behavior of colloid carcinoma in the breast and pancreas. Am J Surg Pathol 27(5):571–578. 10.1097/00000478-200305000-00002 12717243 10.1097/00000478-200305000-00002

[14] Adsay NV, Merati K, Basturk O et al (2004) Pathologically and biologically distinct types of epithelium in intraductal papillary mucinous neoplasms: delineation of an intestinal pathway of carcinogenesis in the pancreas. Am J Surg Pathol 28(7):839–848. 10.1097/00000478-200407000-00001 15223952 10.1097/00000478-200407000-00001

[15] World Health Organization. International Classification of Diseases for Oncology (ICD-O). 3rd ed., 1st revision. World Health Organization (2013) Accessed May 1, 2024. https://iris.who.int/handle/10665/96612

[16] Orcutt ST, Coppola D, Hodul PJ (2016) Colloid carcinoma of the pancreas: case report and review of the literature. Case Rep Pancreat Cancer 2(1):40–45. 10.1089/crpc.2016.0006 30631814 10.1089/crpc.2016.0006 PMC6319686

[17] Tempero MA, Malafa MP, Al-Hawary M et al (2021) Pancreatic adenocarcinoma, version 2.2021, NCCN clinical practice guidelines in oncology. J Natl Compr Canc Netw 19(4):439–457. 10.6004/jnccn.2021.0017 33845462 10.6004/jnccn.2021.0017

[18] Seufferlein T, Mayerle J, Böck S et al (2022) S3-Leitlinie zum Exokrinen Pankreaskarzinom – Langversion 2.0 – Dezember 2021 – AWMF-Registernummer: 032/010OL. Z Gastroenterol 60(11):e812–e909. 10.1055/a-1856-7346 36368658 10.1055/a-1856-7346

[19] Petruch N, Servin Rojas M, Lillemoe KD et al (2024) The impact of surgical-oncologic textbook outcome in patients with stage I to III pancreatic ductal adenocarcinoma: a cross-validation study of two National registries. Surgery 175(4):1120–1127. 10.1016/j.surg.2023.11.004 38092633 10.1016/j.surg.2023.11.004

[20] Chikhladze S, Lederer AK, Kousoulas L et al (2019) Adjuvant chemotherapy after surgery for pancreatic ductal adenocarcinoma: retrospective real-life data. World J Surg Oncol 17(1):185. 10.1186/s12957-019-1732-3 31706323 10.1186/s12957-019-1732-3 PMC6842534

[21] Conroy T, Hammel P, Hebbar M et al (2018) FOLFIRINOX or gemcitabine as adjuvant therapy for pancreatic cancer. N Engl J Med 379(25):2395–2406. 10.1056/NEJMoa1809775 30575490 10.1056/NEJMoa1809775

[22] Picado O, Dosch AR, Garcia-Buitrago MT, Yakoub D, Dudeja V, Rodgers SE (2021) The role of perioperative chemotherapy in the management of colloid carcinoma of the pancreas. Pancreas 50(3):306–312. 10.1097/MPA.0000000000001787 33835960 10.1097/MPA.0000000000001787

[23] Aronsson L, Marinko S, Ansari D, Andersson R (2019) Adjuvant therapy in invasive intraductal papillary mucinous neoplasm (IPMN) of the pancreas: a systematic review. Ann Transl Med 7(22):689. 10.21037/atm.2019.10.37 31930090 10.21037/atm.2019.10.37 PMC6944598

[24] Mungo B, Croce C, Oba A et al (2021) Controversial role of adjuvant therapy in node-negative invasive intraductal papillary mucinous neoplasm. Ann Surg Oncol 28(3):1533–1542. 10.1245/s10434-020-08916-6 32743713 10.1245/s10434-020-08916-6

[25] Duconseil P, Périnel J, Autret A et al (2017) Resectable invasive IPMN versus sporadic pancreatic adenocarcinoma of the head of the pancreas: should these two different diseases receive the same treatment? A matched comparison study of the French surgical association (AFC). Eur J Surg Oncol 43(9):1704–1710. 10.1016/j.ejso.2017.06.011 28687431 10.1016/j.ejso.2017.06.011

[26] Abdalla TSA, Duhn J, Klinkhammer-Schalke M et al (2024) Oncological outcomes and patterns of recurrence after the surgical resection of an invasive intraductal papillary mucinous neoplasm versus primary pancreatic ductal adenocarcinoma: an analysis from the German cancer registry group of the society of German tumor centers. Cancers 16(11):2016. 10.3390/cancers16112016 38893136 10.3390/cancers16112016 PMC11171342

