# Supplementary material for: Oncologic outcomes and prognostic factors of colloid carcinoma of the pancreas – a retrospective real-world data analysis from the German cancer registry group of the society of German tumor centers
Source: Langenbecks Arch Surg. 2025 Sep 23;410(1):275. doi: 10.1007/s00423-025-03870-x (PMC12457452; doi:10.1007/s00423-025-03870-x)
Supplement: Supplementary file 1 — (DOCX 20.6 KB) [file 423_2025_3870_MOESM1_ESM.docx]

**Supplementary Tables**

**Supplementary Table 1a: Survival Analysis of Epidemiological Factors in resected CC and PDAC-NOS patients.** The table comparing the Overall Survival of resected CC and PDAC-NOS patients. Displayed is the median OS from diagnosis in months with the 95 % Confidence interval. Statistical differences were computed by Log-rank test.

|  | **CC** | | | | **PDAC-NOS** | | | |
| --- | --- | --- | --- | --- | --- | --- | --- | --- |
| **Parameter** | **N** | **Events** | **Median survival in months (95% CI)** | **p-value** | **N** | **Events** | **Median survival in months (95% CI)** | **p-value** |
| **Overall** | 433 | 333 | 24.8 (22.3 – 27.6) |  | 20159 | 16415 | 17.3 (16.9 – 17.7) |  |
| **Sex** |  |  |  | 0.73 |  |  |  | 0.0012 |
| male | 259 | 200 | 24.8 (21.7 – 28.7) |  | 10533 | 8655 | 16.7 (16.4 – 17.1) |  |
| female | 174 | 133 | 24.8 (20.7 – 29.8) |  | 9625 | 7760 | 17.9 (17.5 – 18.3) |  |
| **Age (years)** |  |  |  | 0.014 |  |  |  | <0.001 |
| <65 | 130 | 95 | 29.5 (24.8 – 33.9) |  | 6372 | 5014 | 20.6 (19.8 – 21.1) |  |
| ≥65 | 303 | 238 | 22.5 (18.8 – 25.7) |  | 13787 | 11401 | 15.7 (15.5 – 16.2) |  |

**Supplementary Table 1b: Survival Analysis of Histopathological Factors in resected CC and PDAC-NOS patients.** The table comparing the Overall Survival of resected CC and PDAC-NOS patients. Displayed is the median OS from diagnosis in months with the 95 % Confidence interval. Statistical differences were computed by Log-rank test.

|  | **CC** | | | | **PDAC-NOS** | | | |
| --- | --- | --- | --- | --- | --- | --- | --- | --- |
| **Parameter** | **N** | **Events** | **Median survival in months (95% CI)** | **p-value** | **N** | **Events** | **Median survival in months (95% CI)** | **p-value** |
| **T-stage** |  |  |  | 0.16 |  |  |  | <0.001 |
| pT0 | 1 | 1 | 49.4 (NR – NR) |  | 39 | 19 | 45.5 (30.7 – NR) |  |
| pT1 | 31 | 18 | 39.4 (27.4 – NR) |  | 1251 | 730 | 29.7 (27.6 – 32.2) |  |
| pT2/3 | 372 | 292 | 24.1 (20.6 – 25.9) |  | 17876 | 14811 | 16.8 (16.7 – 17.3) |  |
| pT4 | 16 | 13 | 22.2 (15.3 – NR) |  | 612 | 543 | 12.2 (10.6 – 13.4) |  |
| **Lymph node metastases** |  |  |  | <0.001 |  |  |  | <0.001 |
| N0 | 175 | 116 | 39.6 (32.0 – 46.7) |  | 6100 | 4512 | 32.2 (22.4 – 23.9) |  |
| N+ | 242 | 205 | 18.7 (16.6 – 22.7) |  | 13607 | 11531 | 15.5 (15.2 – 15.7) |  |
| **Lymph vessel invasion** |  |  |  | <0.001 |  |  |  | <0.001 |
| L0 | 167 | 110 | 34.9 (29.5 – 44.1) |  | 6996 | 5229 | 20.7 (20.1 – 21.5) |  |
| L1 | 150 | 125 | 18.7 (15.3 – 24.1) |  | 9004 | 7476 | 15.5 (15.0 – 15.7) |  |
| **Blood vessel invasion** |  |  |  | <0.001 |  |  |  | <0.001 |
| V0 | 259 | 187 | 27.6 (24.4 – 32.3) |  | 11869 | 9196 | 19.1 (18.7 – 19.6) |  |
| V1 | 44 | 35 | 14.8 (10.3 – 25.6) |  | 3821 | 3236 | 13.4 (12.8 – 13.7) |  |
| **Grading** |  |  |  | 0.005 |  |  |  | <0.001 |
| G1/2 | 259 | 196 | 27.2 (24.2 – 33.6) |  | 10498 | 8300 | 20.6 (20.0 – 21.0) |  |
| G3/4 | 125 | 105 | 20.0 (15.7 – 24.1) |  | 8129 | 6872 | 13.9 (13.7 – 14.4) |  |
| **Resection margins** |  |  |  | <0.001 |  |  |  | <0.001 |
| R0 | 218 | 154 | 28.9 (24.8 (34.0) |  | 10519 | 7852 | 20.4 (19.8 – 20.8) |  |
| R+ | 84 | 70 | 20.0 (15.9 – 24.8) |  | 4291 | 3712 | 12.6 (12.2 – 12.9) |  |
