# Supplementary material for: Oncologic outcomes and prognostic factors of colloid carcinoma of the pancreas – a retrospective real-world data analysis from the German cancer registry group of the society of German tumor centers
Source: Langenbecks Arch Surg. 2025 Sep 23;410(1):275. doi: 10.1007/s00423-025-03870-x (PMC12457452; doi:10.1007/s00423-025-03870-x)
Supplement: Supplementary file 2 — (DOCX 744 KB) [file 423_2025_3870_MOESM2_ESM.docx]

**Supplementary Figures**

**
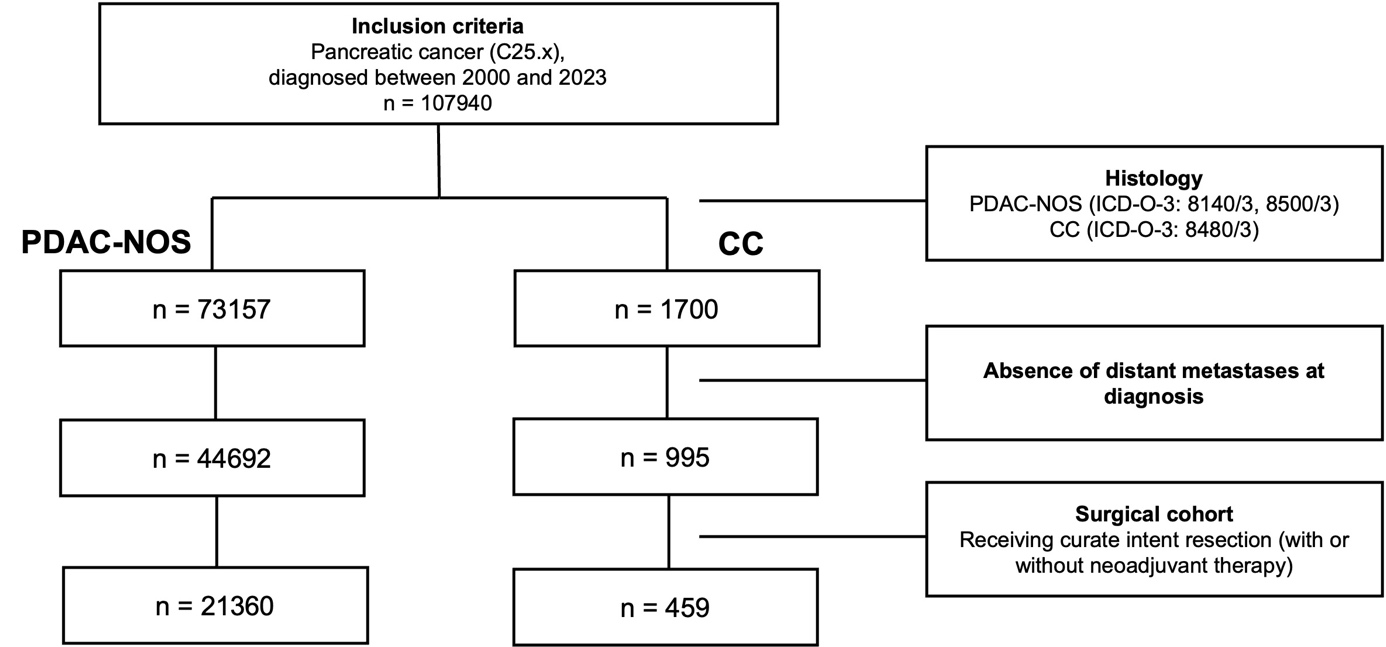
**

**Suppl. Fig. 1: Consort diagram.**

**
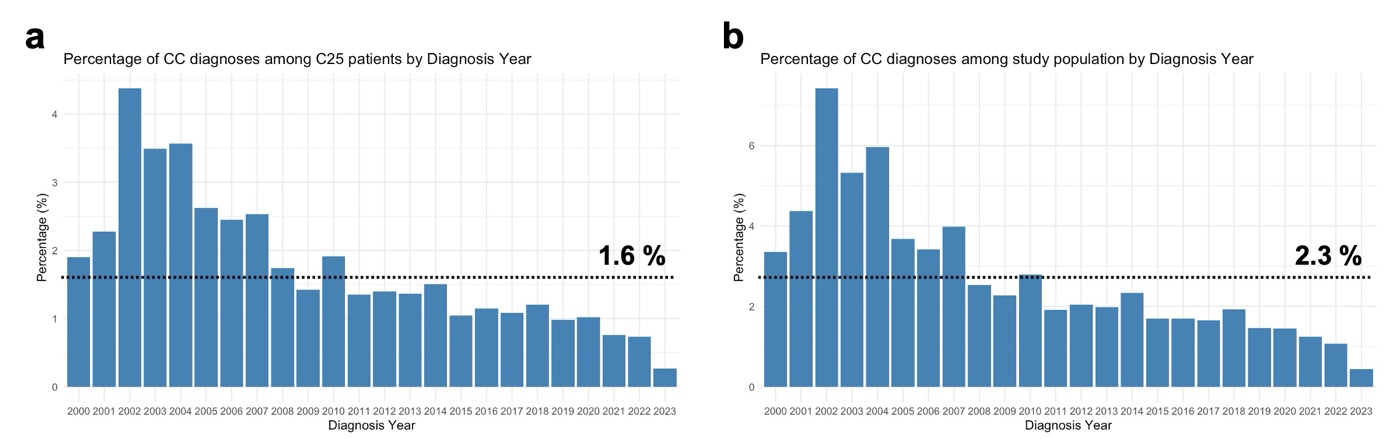
**

**Suppl. Fig. 2: Percentage of CC diagnoses over the study period.** Shown are the percentages of CC diagnoses over the study period from 2000 – 2023 among all patients with malignant tumors of the pancreas (ICD-10: C25; **a**) and the study population consisting of patients with PDAC-NOS or CC (**b**). The dotted line represents the overall percentage within the study period.

**
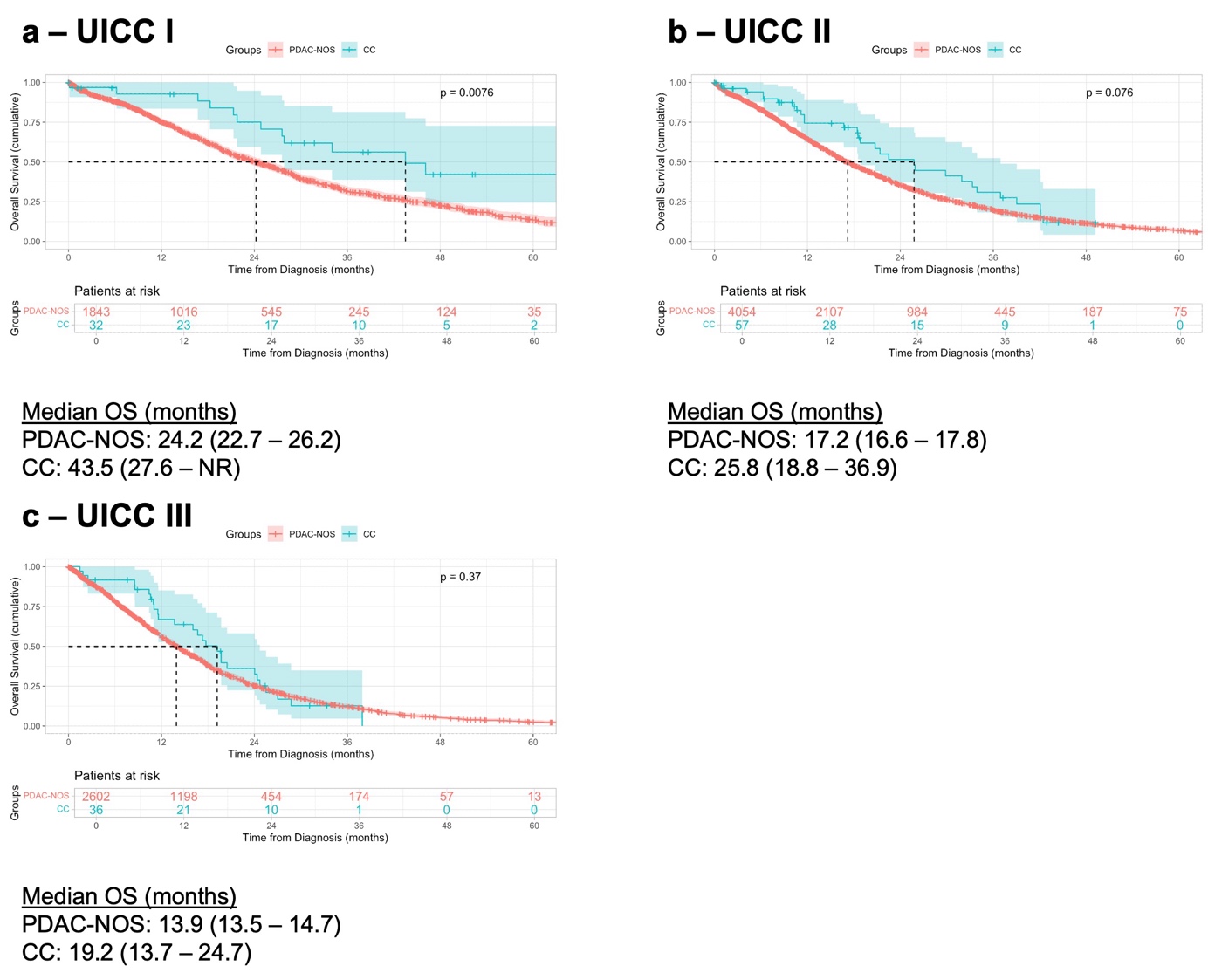
**

**Suppl. Fig. 3: Survival analysis in Stage-matched cohorts.** Kaplan-Meier curves showing the overall survival from diagnosis in months with 95% Confidence interval between resected PDAC-NOS or CC patients stratified according to UICC I **(a)**, UICC II **(b)**, and UICC III **(c)** stages. Only patients with 8^th^ edition AJCC-staging were identified to ensure comparability. The displayed p-values were calculated using the Log-rank test.

**
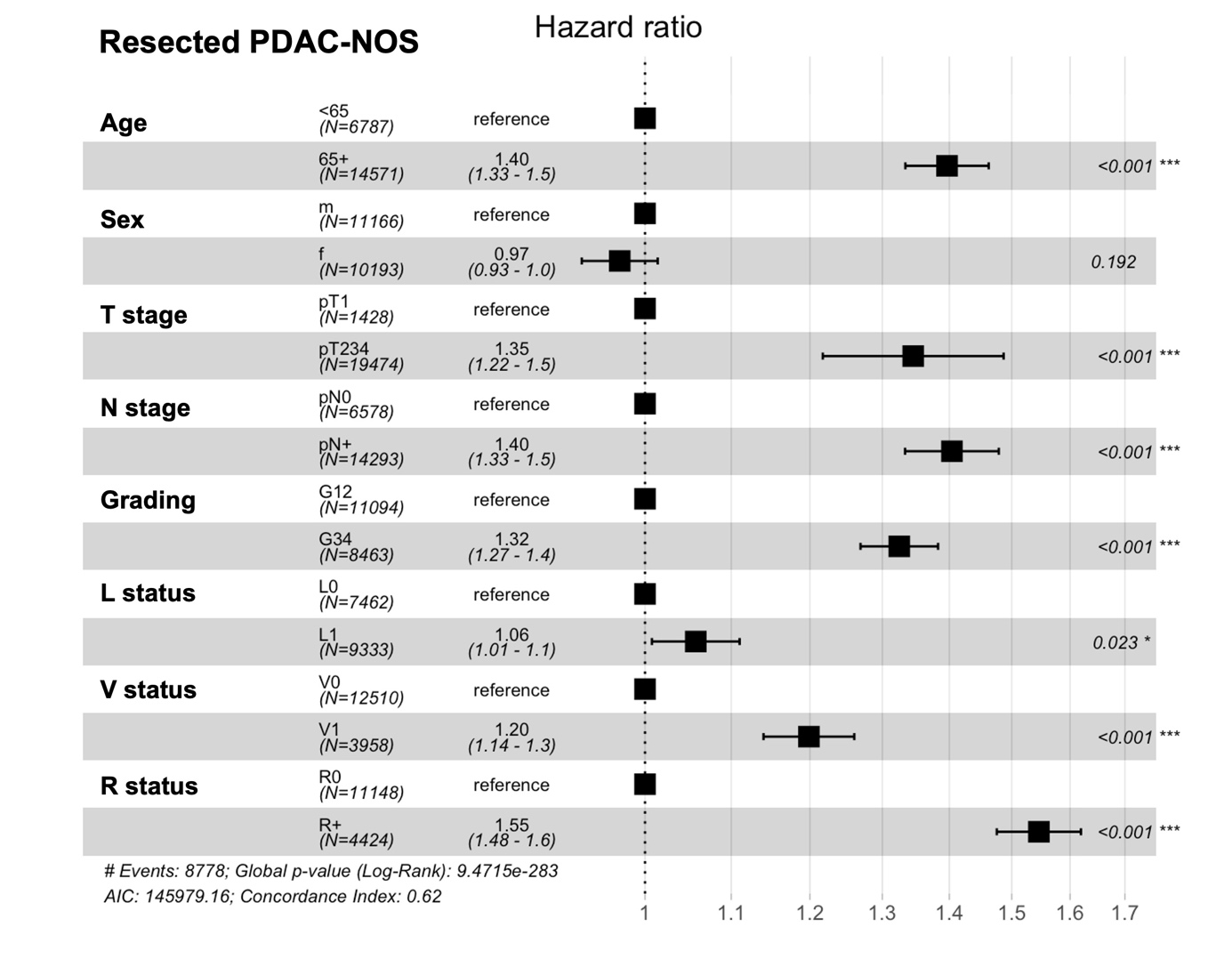
**

**Suppl. Fig. 4: Multivariable Analysis of Prognostic Factors in resected PDAC-NOS patients.** Multivariable cox-regression analysis of the overall survival of resected PDAC-NOS patients. Shown are the hazards ratios with the 95% Confidence Interval, as well as the p-values.
